# Infectious disease and economics: The case for considering multi-sectoral impacts

**DOI:** 10.1016/j.onehlt.2018.100080

**Published:** 2019-01-09

**Authors:** Kristine M. Smith, Catherine C. Machalaba, Richard Seifman, Yasha Feferholtz, William B. Karesh

**Affiliations:** a EcoHealth Alliance, 460 West 34th St, 1701, New York, NY 10001, USA; b City University of New York Graduate School of Public Health & Health Policy, 55 West 125th Street, New York, NY 10027, USA; c United Nations Association-National Capital Area, Washington, DC 20433, USA; d Working Group on Wildlife, World Organisation for Animal Health, 12 Rue du Prony, Paris 75017, France

**Keywords:** One Health, Economic, Infectious disease, Preparedness, Zoonoses

## Abstract

Beyond the public health impacts of regional or global emerging and endemic infectious disease events lay wider socioeconomic consequences that are often not considered in risk or impact assessments. With rapid and extensive international travel and trade, such events can elicit economic shock waves far beyond the realm of traditional health sectors and original geographical range of a pathogen. While private sector organizations are impacted indirectly by these disease events, they are under-recognized yet effective stakeholders that can provide critical information, resources, and key partnerships to public and private health systems in response to and in preparation for potential infectious disease events and their socioeconomic consequences.

## Highlights

• Assessing multi-sector impacts of zoonotic disease events provides new insights beyond engagement of the human health sector.
• The private sector is an under-recognized stakeholder with key information and resources.
• Public and private sector collaboration advances risk analysis and cost-sharing strategies.

## 1 Introduction

Health disasters such as the Ebola virus disease epidemic in West Africa, the Middle East Respiratory Syndrome (MERS) outbreak in the Republic of Korea, and the rise of antimicrobial-resistant pathogens, have catalyzed investments in global health security. As the public health community works to strengthen national systems to avoid international spread of disease, governing bodies increasingly recognize that biological threats not only have global health impacts but also wide-ranging socioeconomic disruptions [1]. More comprehensive economic assessments can provide a multi-sector translational understanding of the costs of disease beyond traditional human health-centric approaches that only consider cases of disease, direct medical spending, and public health functions and interventions (herein collectively defined broadly as the “health sector”).

Health is core to a thriving, productive society, whereas fear and illness can stifle production, consumption, recreation, travel, and overall well-being. While sectors outside of health are often considered in the context of negative externalities in driving disease events, the potential impacts they face from disease events warrants their engagement in finding multi-sectoral solutions to reduce and manage disease risks. On a broad scale, far-reaching impacts of pandemics parallel other disasters. The Ebola epidemic in West Africa demonstrated the serious, and unanticipated, economic toll of an emerging infectious disease. From 2013 to 2014, Gross Domestic Product (GDP) growth in Liberia decreased from 8.7% to 0.7%, due to Ebola and lowering commodity prices, and GDP growth in Sierra Leone (excluding iron ore) decreased from 5.3% to 0.8% [2]. GDP growth in Guinea in 2015, predicted at 4%, fell to 0.1%. In all three countries, government revenues declined across the board, including direct taxes on companies, VAT receipts, and indirect taxes; Additionally, decline in private and foreign investors' confidence led to financing gaps of more than US $600 million over the two years [2]. These impacts cut across many sectors and undoubtedly have long-term consequences, including implications for insurers and reinsurers (e.g., health, life), as well as overall business continuity from lack of worker capacity during illness [3], and markets are emerging to insure against pandemic risk (such as the World Bank's Pandemic Emergency Financing Facility, a parametric insurance vehicle designed to provide rapid disbursement of emergency finance [4]). Yet engagement of the private sector, as well as public institutions beyond the health sector, remains limited in overall epidemic and pandemic planning and intervention.

Greater appreciation of the economy-wide impacts of epidemics (i.e. to determine macro-economic trends towards a general equilibrium model, rather than effects on only one sector or market) is warranted. We present examples from the literature illustrating the breadth of impacts from recent epidemics to demonstrate that a whole-of-society approach is warranted to address infectious disease risk. Broadening engagement beyond the health sector can help shape the direction of initiatives like the Global Preparedness Monitoring Board jointly launched by the World Bank and World Health Organization in 2018 to monitor response readiness for pandemics and other health emergencies [5].

## 2 Materials and methods

### 2.1 Economic impact assessments of disease (and relevance of broad stakeholder engagement)

The conventional scope of estimating economic impact of disease events in humans has often been limited to basic direct costs (health care) and limited indirect losses (e.g., wages not earned and informal health costs such as patient transport). Disease burden may be captured in health metrics (e.g., number of deaths or Disability-Adjusted Life Years). While meaningful for the health community, it is increasingly becoming evident that this limited scope of analysis does not provide a comprehensive view of economic consequence of disease events, including contagion avoidance behaviors, to inform decision-making by a wider range of stakeholders and connect to broader economic development agendas. Direct and indirect economic impacts of disease events are affected by disease preparedness and prevention (practices that mitigate risk), the event itself (e.g., business continuity, supply chain disruption, trade and travel bans, public contagion avoidance behavior), and the event aftermath (e.g., long-term employment loss, permanently closed markets or farms, long-term stigmas associated with specific animal products, impacts of childhood lost education or being orphaned, etc.).

In 2016, the Second Panel on Cost-Effectiveness in Health and Medicine highlighted the importance of applying the societal perspective beyond only a health sector perspective when considering economic impacts of disease as well as potential interventions [6]. Similarly, the World Health Organization (WHO) has proposed an economic impact guide as a framework within which broader economic impact of diseases can be calculated [7], and a proposed framework to analyze economic consequences of biothreats has been developed that examines broader impacts, including human behavior, speed of resilience, and the “fear factor” dynamic that may cause irrational behaviors aimed at disease avoidance [8]. These are preceded by an extensive body of work from the animal health sector that thinks of disease as an issue beyond the veterinary sector, emphasizing the need for assessing economic impacts throughout a system to be inclusive of also the costs of expenditure and reaction to a disease outbreak or disease presence in addition to direct costs (in the case of livestock, for example, accounting for not just production but also productivity, and costs that are ‘invisible’ and thus often unaccounted for, such as effects on herd structure from diminished calving rates, effects on market access, animal and human welfare, etc.) [[9], [10], [11]] Impacts may be incurred upstream or downstream; understanding these different implications can therefore help to determine total costs of diseases and optimize the net benefits of decisions taken for prevention and control [12,13].

The World Bank has reported significant costs of diseases that occur at the human-animal-environment interface and necessitate a One Health approach, with a high global return value for investing in prevention through strengthening veterinary and human health capacity [14]. Taking multi-sectoral approaches to disease risk reduction and management can inform on possible economic outcomes (positive or negative) that may be incurred to any given sector(s) from various prevention and control strategies [15].

We illustrate cases where indirect impacts of infectious disease impacts have been significant. Though such impacts have been rarely appreciated to date, the recognition of nontraditional stakeholders in biological threat impact analysis advocates for their investment and involvement in risk assessment, preparedness, and/or intervention efforts. In particular, these can help inform stakeholders for inclusion in multi-sectoral national action plans for health security and similar budgeting processes that engage finance ministries and legislative bodies that can allocate resources to optimize ‘whole-of-society’ outcomes.

### 2.2 Examples of multi-sectoral impacts of infectious disease outbreaks

#### 2.2.1 Health sector

Health sector impacts of infectious disease outbreaks are often the most straightforward to estimate or at least tally retroactively. However, for novel or reemerging pathogens with unexpected clinical outcomes, predictions can be difficult and cost estimates are frequently limited to short-term medical spending, health burden, or mortality. For example, while typical Zika infections without sequelae are unlikely to result in significant burden, disease manifestation in infants can have extensive impacts. Not only are direct medical expenses expected to build during pregnancy, but post-natal direct and indirect costs - particularly given the implied long-term extensive care required for these children as they grow - will be significant. Alfaro-Murillo et al. [16] conservatively estimated the lifetime direct medical cost associated with sequelae to microcephaly to be US $179,760, and the costs per case of Gillian-Barre Syndrome, a rare outcome of Zika cases (roughly 1%), to be US $56,863, in the US. Ulansky estimated that severe cases of GBS could cost up to US $500,000 per year and that lifetime health costs of microcephaly affected children could reach US $10 million each [17]. These estimates would vary by country, and do not include indirect costs such as specialized child-care support, parental productivity losses, psychological toll on families with children with microcephaly, or losses in productivity of the child once an adult and the support services required for that individual throughout life [16]. It was estimated the Zika epidemic thus far has cost Latin America and the Caribbean US $7-18 billion from 2015 to 2017 alone [18].

The 2013–2015 Ebola crisis in West Africa resulted in at least 28,616 suspected cases and 11,310 confirmed deaths [19]. In comparison, there had been 2427 cases and 1597 deaths in all other known outbreaks of Ebola combined [20]. The breadth and depth of the crisis was intensified due to poor health care systems in the nations it impacted. The outbreak led to 881 infections in healthcare workers themselves (513 deaths). The entire healthcare workforce declined by 8% in Liberia, and 23% in Sierra Leone; this loss in healthcare services led to an estimated 10,600 additional deaths due to untreated conditions in Guinea, Liberia, and Sierra Leone (1091 deaths due to HIV, 2714 deaths due to tuberculosis; and 6818 deaths due to malaria) [20,21]. Further, prenatal consultations declined, out-of-hospital child births increased (in-hospital and health clinic births dropped by 30% in Sierra Leone), childhood vaccination coverage decreased 30% during the outbreak, and childhood deaths increased from measles and other vaccine-preventable diseases [20].

#### 2.2.2 Agricultural sector/food animal production systems

Given that 60% of all human infectious pathogens originate from animals [22], agricultural sectors involved in zoonotic outbreaks often suffer significant economic impacts that are underappreciated. Fifty percent of reported livestock losses to the World Organisation for Animal Health (OIE), the international standard-setting organization for livestock disease, are due to zoonoses, and zoonoses have a much higher percentage of animal slaughter (43% of livestock losses) as part of disposal for disease control compared to non-zoonotic events (6% of livestock losses) [14]. Yet the incentive for the agricultural sector (i.e., food animal production) to invest in infectious disease prevention often correlates with the economic relevance of the industry to its overall national GDP. For example, in the US where net meat exports near 12% of production [23], investments in animal health infrastructure are prioritized. However, many developing countries that engage in agricultural trade have competing priorities resulting in lower investment in animal health infrastructure and protection, and thus may not employ adequate biosecurity measures. After Saudi Arabia and Yemen suffered an introduction of Rift Valley fever virus in 2000, Arabian countries banned imports of live animals from at least nine African countries, causing the Somalian livestock market to completely collapse. Ninety percent of Somalia's total income had been from livestock export, and the ban resulted in a loss of over 75% in exports and US $300 million [24]. This caused social and financial instability, loss of livelihoods and food security, and ultimately instability of the Somalian government, with a 25–36% reduction in GDP [24].

Costs of infectious disease outbreaks to the agriculture sector are often measured in value of culled livestock alone, while wider long-term impacts remain under-recognized. During the 1998 Nipah virus outbreak in Malaysia (resulting in 283 human cases of viral encephalitis and 109 fatalities), the Malaysian government paid US $97 million in compensation for the 1.1 million pigs culled due to the outbreak. But beyond that, these impacts led to an additional US $229 million in indirect costs in lost tax revenue to government and losses in international trade, and a US $136 million cost for a control program for biosecurity and slaughter facilities [25]. Pork consumption and exports remained altered long-term (dropping by 80% during the outbreak and remaining 30% depressed post-outbreak; [26]). Unmeasured economic impacts of this outbreak in Malaysia continue to this day. The pig farming industry in hard hit areas collapsed, forcing many pig farmers to attempt to transition to other jobs for which they had no training or education. Long-term unemployment or underemployment ensued for these families and they have been unable to reach their previous economic status, also affecting the many local businesses that thrived upon them [27].

In the case of H1N1 pandemic influenza in Mexico, the mere public perception of risk resulted in costly consequences for the country's swine production industry; exports of chilled and fresh pork saw drastic declines (e.g., a reduction of >60% to Japan), resulting in the country's pork trade deficit of US $27 million by the end of 2009 [28].

#### 2.2.3 Tourism and travel

During the 2003 SARS outbreak, tourist arrivals in Hong Kong dropped 68% just two months after the WHO issued a warning about the epidemic; Asia-Pacific carriers saw a US $6 billion loss in revenue and North American airlines saw another US $1 billion loss [29]. Singapore tourism fell >70%, causing Singapore Airlines to place 6600 flight staff on unpaid leave [30]. Effects were also acutely felt at local levels. For example, China's Guangzhou trade fair saw 12% attendance compared to previous year [31].

In South Korea, where an introduction of MERS caused a brief 2015 outbreak, the number of international visitors dropped by 41% in mid-summer compared with the same month the prior year. These visitors decreased a further 60% only one month later. The Korean government lost US $10 billion [32], and had to put forth costly tourism campaigns the following years to encourage travelers to visit. Similarly, Saudi Arabia's tourism industry was impacted an estimated US $5 billion per year due to MERS-related travel restrictions.

The H1N1 influenza resulted in a US $2.8 billion hit to Mexico's tourism industry, its largest service sector, with a loss of one million tourists over a five-month period due to contagion fears [28]. Zika virus has displayed similar tendencies in self-restricted travel by consumers nervous over exposure. Concerns and travel warnings in affected regions of the world provide brief insight. Even limited travel advisories in the high-tourism area of Miami, Florida, US caused political and economic backlash, with businesses reportedly seeing a 50–60% loss of revenue [33]. If the virus continues to spread in nations in which tourism is a key component of the GDP, such as the Caribbean nations, the economic impact of this disease on the travel industries will likely rise significantly.

#### 2.2.4 Trade and retail industries

After killing at least 800 people and infecting >8000, the total global economic loss due to SARS was estimated to near US $40 billion [14,34]. The Chinese bureau of statistics reported a 0.8% loss in GDP in 2003, mainly comprised of losses to the tourism, travel, hotel, restaurant, and retail industries [34]. Much of this impact was due to consumer fears given the ease of transmissibility of the virus in public settings. Hong Kong took a 2.6% hit to its GDP [34]. International transport companies such as FedEx and airport shops such as Estée Lauder were impacted, sending economic repercussions across the globe. Transportation restrictions and cancellations impacted multi-national industries such as oil, for which demand fell by 300,000 barrels a day in Asia [29].

The wider economic impact of the 1998 Nipah outbreak in Malaysia was estimated at US $582 million [25,35]. Losses affected sectors indirectly related to the pork industry (e.g., the feed industry that supplied nutrition to pigs saw an estimated US $15 million reduction in its production; [26]). Approximately 618 homes, 111 shops, schools and banks were evacuated and an estimated 36,000 people lost their jobs not only from within the pork industry but from a wide range of business activities such as utility and the real estate industry (which lost US $1.1 million) [35].

During the South Korean MERS outbreak, the public's contagion fear and governmental overreaction closed down many public events and stifled daily activities [32]. The accommodation and food sectors experienced a 10% drop in production from the previous year; the entertainment and recreation sector production likewise dropped 8.6%, and publishing, communication and information sectors dropped 6.3% [32]. Transportation and storage dropped 2.4%, wholesale and retail dropped 1.6%, and electricity and air conditioning 0.9% [32]. However, market response indicated behavior change due to avoidance of public settings: South Korea's largest market chain, *E*-Mart Co Ltd., reported online sales rose 63%, and for the second largest chain, Homeplus, online sales rose 50% in early June due to consumers avoiding brick-and-mortar stores [36]. Meanwhile, the industries with high proportions of temporary jobs (e.g. restaurant, accommodation, and recreation sectors), who are also typically disproportionately affected by outbreaks, were significantly impacted, leading to labor losses [37]. South Korean exports were also affected, with the economy only growing 0.3% in the second quarter of 2015; a six-year record low.

#### 2.2.5 Environmental impacts

As environmental resources and services are typically considered non-market goods (i.e., they are not traded in markets or their market price does not reflect their true value), damage to valuable natural resources, loss of wildlife populations, and contamination of the environment are often overlooked in economic assessments relating to disease events. Local demand for natural resources may rise during socioeconomic and stability crises, leading to increased wildlife harvest and illegal use of protected lands; enforcement of environmental protection policies may decline when the government is overwhelmed with other burdens [i.e. 38,39]. For example, the quarantine and travel restriction measures and enforcement during the West Africa Ebola outbreak led to illegal poaching, logging, and mining and “negatively impacted advances made in protecting water catchment areas, forest and animal reserves, thus reversing efforts made previously towards attaining the Millennium Development Goals related to environmental protection” [40]. During the H5N1 influenza outbreaks national authorities conducted wild bird culling, closed protected wetlands, and destroyed waterbird habitats in misguided attempts to halt the virus spread [41].

Economic tolls of environmental impacts are rarely measured due to poor valuation of ecosystem services and other natural resources; however, focus on environmental value, at least for individual ecosystem services (e.g., pollination) has progressed over the past two decades through initiatives such as The Economics of Ecosystems and Biodiversity [42]. Still, however, they remain extremely limited in their integration with health sector decision making.

#### 2.2.6 Other impacts

While morbidity and mortality values may indicate severity of impact of a disease on a population, they do not allow appreciation of the full consequence of impaired productivity from illness for a person, their household or their community. For example, impacts may involve psychological, educational, or professional losses on the individual consumer and household. Not only did the unusually high death toll during the West Africa Ebola outbreak result in expanded social and household economic impacts, but also in stifled growth rates, lost productivity and wages due to inability to work or contagion fear, increased poverty and food insecurity, lost jobs, and lost education [1]. The extent and type of household economic impacts is often (although not exclusively) related to the population cohort most affected. For Ebola, the age group of 15–44 years, those engaged in the labor force and parents of young children, accounted for 57% of all infections, explaining why the impact on economic activity, poverty and food security was so substantial [1]. Sixty to 70% of households reported their incomes dropped significantly during the outbreak; consumption by households decreased and the prevalence of undernutrition rose [1]. Further, approximately 16,000 children lost parents to Ebola, leaving them orphans needing long-term care by relatives or other means [43]. The closure of schools, resulting in over 33 weeks of lost education, was further believed to have exposed children to several types of child abuse (including sexual exploitation and violence against young girls) with long-term impacts such as emotional trauma, permanent removal from education system, and unwanted pregnancies [44]. Military personnel were drawn from regular public safety duties to enforce quarantine facilities, a task for which they were not trained. These represent only partial examples of the ripple effects at the individual and household level that may impact public and private sectors in myriad short and long-term ways.

### 2.3 Non-traditional stakeholder involvement in assessment and preparedness

Considering both direct and indirect economic impacts of infectious disease events in cost analyses or assessments requires engagement of relevant and impacted sectors. There may be difficulty in isolating, attributing, measuring and comparing indirect losses (such as animal production, travel, recreational, or educational impacts), as well as expenses incurred due to public messaging, transportation disruptions or policy changes, surveillance, or biosecurity measures for staff. Furthermore, while a disease event may negatively impact one sector, another can potentially benefit. One must decide which sectors to include in assessments and how far to extend them temporally and geographically. However, comparisons on a country level may be practical and actionable to guide decision-making on budgets, regulations, and agency mandates.

Stakeholders will vary based on specific disease event, its scope and range of impacts. Economic implications of unpredicted infectious disease events can be detrimental not only to public health systems but to food and agriculture industries, trade and travel, various market types and retail chains, mining, oil and gas and natural resource providers, environment and ecosystem services, among others (Table 1). These sectors have not traditionally been directly involved in disease impact assessments or preparedness planning (including prevention efforts), yet they have increasingly recognized the threat of health disasters wherein consumers are too fearful or unable to access their services because of supply chain or other business continuity impact, or their workforce is directly compromised. Inclusion of relevant non-health stakeholders in risk and impact assessments may provide more informed health impact assessments and enhanced awareness regarding preparedness opportunities, and may provide access to new collaborations and potential risk mitigation and resources. For example, the agricultural industry can promote strong biosecurity practices along their supply chains, the pharmaceutical industry can improve regulatory mechanisms or guidelines to discourage antibiotic resistance, utilities sectors can encourage water sourcing methods that minimizes health threats [45] and energy and extractives sectors can ensure a safe, reliable protein source for employees to reduce risky wildlife hunting practices that may be associated with natural resource development.

**Table 1:** Examples of financial impacts due to zoonotic infectious disease events beyond the public health sector.

Sectors impacted	Time period	Geographic scope	Disease	Metrics	Economic estimate	References
Tourism	2009	Mexico	H1N1	tourism	2.8 billion	[28]
Agriculture	1998–2002	Somalia	RVF	livestock export losses	435 million	[51]
Government	1998–1999	Malaysia	Nipah	lost tax revenue	105 million	[35]
Financial	2013–2015	Ghana, Liberia, Sierra Leone	Ebola	loss of investor confidence	600 million	[2]
Travel	2003	Global	SARS	airline losses	7 billion+	[29]

The private sector is often highly motivated to quell consumer fears and avoid profit losses, and is dependent upon employee and customer health. Thus, it may meaningfully contribute to preparedness and response, as shown by the Ebola Private Sector Mobilisation Group in West Africa that was established to facilitate a coordinated private sector response [46]. The group focused on educating and advocating for their employees and providing economic stability. They reported donating “personnel, equipment, building infrastructure and…provided expertise such as construction, logistics, distribution services…facilitated connections between companies and support organisations.” For example, ArcelorMittal and Firestone created community awareness and screening programs and contributed machinery and capacity to construct isolation and treatment centers [47].

National governments and responding agencies may have to learn how to coordinate with private companies and understand their contribution potential. Conducting risk analyses and preparedness with these sectors prior to such events would help facilitate potential pathways for involvement in outbreak response, or ideally, to help reduce disease risks upstream to drive benefits downstream (for example, effective preparedness and initial response has been found to affect quality and cost-effectiveness of follow-on disease control in the animal health sector; this may require upfront investments in risk reduction, i.e. biosecurity) [12,48].The World Economic Forum has created recommendations for public-private cooperation models to manage any potential future outbreaks more effectively and reduce the risk of their occurrence [47]. Risk reduction guidelines for specific industries, whether taken up voluntarily or built into donor or private financing mechanisms, may also help with longer-term disease prevention or management; for example, audit and planning tools targeted at reducing risk of emerging infectious diseases have been developed for extractive industries [49].The value of risk reduction aligns with the recent inclusion of ‘Disease X' on the WHO's R&D Blueprint, acknowledging that the next epidemic could be caused by a pathogen currently unknown or unexpected. The wide impacts- both proven and potential- of known and unknown diseases warrants assurance that disease risks, responses and recognition of impacts are not relegated to only the health sector.

## 3 Discussion

An analysis conducted by the World Bank estimates the economic losses from six major outbreaks of highly fatal zoonoses between 1997 and 2009 amounted to at least US $80 billion. If these outbreaks had been prevented, the avoided losses would have averaged US $6.7 billion per year [14]. The wide-ranging – and often substantial - economic impacts of epidemics are increasingly recognized far beyond the health sector. Yet few studies apply a One Health or multi-sectoral lens to consider costs and benefits of prevention versus response effort during planning exercises to ensure optimization of resources. With recent zoonotic disease prioritization exercises being conducted under the Global Health Security Agenda, countries have an opportunity to consider the focus and scope of their investments. Where possible, investments should seek to strengthen overall human, animal and environmental health systems for multi-hazard preparedness and broad societal benefits. Availability of quantitative impact data has been noted as limited for important livestock diseases, and differing methodologies result in estimates that are not comparable across and even within countries [12,48,50]; the findings of our review are consistent with this and also suggest that impacts of human health emergencies from infectious disease are significant but reporting is ad hoc and likely incomplete. Public and private stakeholders at local, national and international levels must work together more systematically to ensure informed systems and risk and impact analysis, and encourage cost-sharing strategies for prevention and preparedness where possible and assess optimal intervention strategies when necessary. Infectious disease events in today's globalized world will require nothing less than such robust public-private partnerships and responsibility for optimal health and economic security.

## Declarations of interest

None.

All authors have approved the final contents of this article.

## References

[1] United Nations Development Group – Western and Central Africa (2015). Socio-Economic Impact of Ebola Virus Disease in West African Countries.

[2] World Bank (2016). 2014–2015 West Africa Ebola Crisis: Impact Update. http://www.worldbank.org/en/topic/macroeconomics/publication/2014-2015-west-africa-ebola-crisis-impact-update.

[3] Lloyd's Emerging Risks Team (2008). Pandemic – Potential Insurance Impacts. https://www.lloyds.com/~/media/lloyds/risk/er_pandemic_insuranceimpacts_v2.pdf.

[4] World Bank (2017). From Panic and Neglect to Investing in Health Security: Financing Pandemic Preparedness at a National Level. http://documents.worldbank.org/curated/en/979591495652724770/pdf/115271-REVISED-FINAL-IWG-Report-3-5-18.pdf.

[5] WHO (2018). WHO and World Bank Group Join Forces to Strengthen Global Health Security. Geneva. https://www.who.int/news-room/detail/24-05-2018-who-and-world-bank-group-join-forces-to-strengthen-global-health-security.

[6] Sanders G.D., Neumann P.J., Basu A., Brock D.W., Feeny D., Krahn M. (2016). Recommendations for conduct, methodological practices, and reporting of cost-effectiveness analyses second panel on cost-effectiveness in health and medicine. JAMA.

[7] World Health Organization (2009). WHO Guide to Identifying the Economic Consequences of Disease and Injury. http://www.who.int/choice/publications/d_economic_impact_guide.pdf.

[8] Rose A.Z. (2009). A framework for analyzing and estimating the total economic impacts of a terrorist attack and national disaster. J. Homel. Secur. Emerg. Manag..

[9] McInerney J. (1996). Old economics for new problems – livestock disease: presidential address. J. Agric. Econ..

[10] Rushton J., Thornton P., Otte M.J. (1999). Methods of economic impact assessment. The Economics of Animal Disease Control.

[11] Rushton J. (2009). The Economics of Animal Health and Production.

[12] Otte M.J., Hinrichs J., Rushton J., Roland-Holst D., Zilberman D. (2008). Impacts of avian influenza virus on animal production in developing countries perspectives in agriculture, veterinary science, nutrition and natural resources. CABI.

[13] Tisdell C. (2009). Economics of Controlling Livestock Diseases: Basic Theory. Rushton, Economics of Animal Health & Production.

[14] World Bank (2012). People, Pathogens and our Planet: The Economics of One Health. https://openknowledge.worldbank.org/handle/10986/11892.

[15] Machalaba C., Smith K.M., Awada L., Berry K., Berthe F., Bouley T.A. (2017). One health, economics to confront disease threats. Trans. R. Soc. Trop. Med. Hyg..

[16] Alfaro-Murillo J.A., Parpia A.S., Fitzpatrick M.C., Tamagnan J.A., Medlock J., Ndeffo-Mbah M.L. (2016). A cost-effectiveness tool for informing policies on Zika virus control. PLoS Negl. Trop. Dis..

[17] Ulansky E. The Economics of Zika. http://thehill.com/opinion/op-ed/284177-the-economics-of-zika.

[18] United Nations Development Programme (2017). A Socio-Economic Impact Assessment of the Zika Virus in Latin America and the Caribbean: With a Focus on Brazil. http://www.undp.org/content/undp/en/home/librarypage/hiv-aids/a-socio-economic-impact-assessment-of-the-zika-virus-in-latin-am.html.

[19] World Health Organization, Ebola outbreak (2014-2015). http://www.who.int/csr/disease/ebola/en/.

[20] Centers for Disease Control and Prevention Cost of the Ebola Epidemic. https://www.cdc.gov/vhf/ebola/pdf/cost-ebola-multipage-infographic.pdf.

[21] Parpia A.S., Ndeffo-Mbah M.L., Wenzel N.S., Galvani A.P. (2016). Effects of response to 2014–2015 Ebola outbreak on deaths from malaria, HIV/AIDS, and tuberculosis, West Africa. Emerg. Infect. Dis..

[22] Taylor L.H., Latham S.M., Woolhouse M.E. (2001). Risk factors for human disease emergence. Philos. Trans. R. Soc. Lond. Ser. B Biol. Sci..

[23] Oklahoma State University, Division of Agricultural Sciences and Natural Resources http://www.dasnr.okstate.edu/Members/donald-stotts-40okstate.edu/global-trade-important-to-all-u-s-meats.

[24] Peyre M., Chevalier V., Abdo-Salem S., Velthuis A., Antoine-Moussiaux N., Thiry E. (2015). A systematic scoping study of the socio-economic impact of Rift Valley fever: research gaps and needs. Zoonoses Public Health.

[25] Dimmock N.J., Easton A.J., Leppard K.N. (2016). Introduction to Modern Virology.

[26] Hosono H., Kono H., Ito S., Shirai J. (2006). Economic impact of Nipah virus infection outbreak in Malaysia, Obihiro University of Agriculture and Veterinary Medicine; National Institute of Animal Health. Proceedings of the 11th International Symposium on Veterinary Epidemiology and Economics.

[27] Ng C.W., Choo W.Y., Chong H.T., Dahlui M., Goh K.J., Tan C.T. (2009). Long-term socioeconomic impact of the Nipah virus encephalitis outbreak in Bukit Pelanduk, Negeri Sembilan, Malaysia: a mixed methods approach. Neurol. Asia.

[28] Rassy D., Smith R.D. (2013). The economic impact of H1N1 on Mexico's tourist and pork sectors. Health Econ..

[29] Begley S. (January 21, 2013). Flu-Conomics: The Next Pandemic Could Trigger Global Recession. https://www.reuters.com/article/us-reutersmagazine-davos-flu-economy/flu-conomics-the-next-pandemic-could-trigger-global-recession-idUSBRE90K0F820130121.

[30] The Economist, Painful Side Effects. http://www.economist.com/node/1747241; http://www.who.int/mediacentre/factsheets/zika/en/ (accessed 18 June 2018).

[31] Bateman J. (May 23, 2016). The Trade Show of Everything. https://www.theatlantic.com/business/archive/2016/05/canton-fair-guangzhou-everything/483545/.

[32] Lee C., Ki M. (2015). Strengthening epidemiologic investigation of infectious diseases in Korea: lessons from the Middle East respiratory Syndrome outbreak. Epidemiol Health.

[33] Chang D., Mazzei P. (August 22, 2016). Florida Governor to Feds: Help us Fight Zika. http://www.miamiherald.com/news/health-care/article97150077.html.

[34] Lee J.W., McKibbin W.J., Knobler S., Mahmoud A., Lemon S., Mack A., Sivitz L., Oberholtzer K. (2004). Estimating the global economic costs of SARS. Learning from SARS: Preparing for the Next Disease Outbreak: Workshop Summary.

[35] FAO (2002). Manual on the Diagnosis of Nipah Virus Infection in Animals. http://www.fao.org/tempref/docrep/fao/005/ac449e/ac449e00.pdf.

[36] Yun Oh S. (2015). Schools Reopen As South Korea Seeks Normality amid MERS Outbreak. http://www.reuters.com/article/us-health-mers-southkorea-idUSKBN0OU14P20150615.

[37] Lee A., Cho J. (2016). The impact of epidemics on labor market: identifying victims of the Middle East respiratory Syndrome in the Korean labor market. Int. J. Equity Health.

[38] Unruh J.D. (2009). Land rights in postwar Liberia: the volatile part of the peace process. Land Use Policy.

[39] Brashares J.S., Golden G.D., Weinbaum K.Z., Barrett C.B., Okello G.V. (2011). Economic and geographic drivers of wildlife consumption in rural Africa. PNAS.

[40] African Development Bank (2014). Government of Sierra Leone, International Monetary Fund, World Bank, UN Development Programme, The Economic and Social Impact of Ebola Virus Disease in Sierra Leone–Joint Preliminary Assessment Report.

[41] (2008). Ramsar 10th Meeting of the Conference of the Parties to the Convention on Wetlands, Resolution X.21. Changwon, Republic of Korea. http://www.fao.org/docs/eims/upload/259339/ak074e00.pdf.

[42] TEEB, Kumar Pushpam (2010). The Economics of Ecosystems and Biodiversity Ecological and Economic Foundations.

[43] World Economic Forum (2016). The Global Risks Report. http://reports.weforum.org/global-risks-2016/global-disease-outbreaks/.

[44] Risso-Gill I., Finnegan L. (2015). Save the Children, Children's Ebola Recovery Assessment: Sierra Leone. http://reliefweb.int/report/sierra-leone/children-s-ebola-recovery-assessment-sierra-leone.

[45] Saker L., Lee K., Cannito B., Gilmore A., Campbell-Lendrum D. (2004). Globalization and Infectious Diseases: A Review of the Linkages. http://www.who.int/tdr/publications/documents/seb_topic3.pdf.

[46] Oxfam America Ebola and the Private Sector Bolstering the Response and West African Economies. https://www.oxfamamerica.org/static/media/files/Ebol-and-the-Private-Sector-oxfam.pdf.

[47] World Economic Forum (2015). Managing the Risk and Impact of Future Epidemics: Options for Public-Private Cooperation. http://www3.weforum.org/docs/WEF_Managing_Risk_Epidemics_report_2015.pdf.

[48] Inamura M., Rushton J., Antón J. (2015). Risk Management of Outbreaks of Livestock Diseases.

[49] United States Agency for International Development Emerging Pandemic Threats-2 Program, Extractive Industry Engagement. https://www.usaid.gov/what-we-do/global-health/pandemic-influenza-and-other-emerging-threats/programs/extractive-industry-engagement.

[50] Rushton J., Gilbert W. (2016). The economics of animal health: direct and indirect costs of animal disease outbreaks. Technical Item Presented at the 84th General Session of the World Assembly of OIE Delegates, 22–27 May, Paris.

[51] Cagnolati V., Tempia S., Abdi A.M. (2006). Economic impact of Rift Valley fever on the Somali livestock industry. International Symposia on Veterinary Epidemiology and Economics Proceedings, ISVEE.

